# Traditional Eastern European diet and mortality: prospective evidence from the HAPIEE study

**DOI:** 10.1007/s00394-020-02319-9

**Published:** 2020-07-01

**Authors:** Denes Stefler, Daniel Brett, Eszter Sarkadi-Nagy, Ewa Kopczynska, Stefan Detchev, Aniko Bati, Mircea Scrob, Diane Koenker, Bojan Aleksov, Elodie Douarin, Galina Simonova, Sofia Malyutina, Ruzena Kubinova, Andrzej Pajak, Milagros Ruiz, Anne Peasey, Hynek Pikhart, Martin Bobak

**Affiliations:** 1 Department of Epidemiology and Public Health, University College London, 1-19 Torrington Place, London, WC1E 7HB UK; 2 School of Slavonic and East European Studies, University College London, London, UK; 3 Department of Nutritional Epidemiology, National Institute of Pharmacy and Nutrition, Budapest, Hungary; 4 Institute of Sociology, Jagiellonian University, Krakow, Poland; 5 Department of History, South-West University, Blagoevgrad, Bulgaria; 6 Institute of Ethnology, MTA Research Centre for the Humanities, Budapest, Hungary; 7 Department of Liberal Arts and Natural Sciences, University of Birmingham, Birmingham, UK; 8 Research Institute of Internal and Preventive Medicine, Branch of the Institute of Cytology and Genetics, Siberian Branch of Russian Academy of Sciences, Novosibirsk, Russia; 9 Novosibirsk State Medical University, Novosibirsk, Russia; 10 National Institute of Public Health, Prague, Czech Republic; 11 Department of Epidemiology and Population Studies, Jagiellonian University Collegium Medicum, Krakow, Poland

**Keywords:** Eastern Europe, Dietary pattern, Mortality, Cardiovascular disease, Cancer

## Abstract

**Purpose:**

Cardiovascular disease (CVD) and cancer mortality rates in Eastern Europe are among the highest in the world. Although diet is an important risk factor, traditional eating habits in this region have not yet been explored. This analysis assessed the relationship between traditional dietary pattern and mortality from all-causes, CVD and cancer in Eastern European cohorts.

**Methods:**

Data from the Health, Alcohol and Psychosocial factors in Eastern Europe prospective cohort were used, including participants from Russia, Poland and the Czech Republic. Based on food frequency questionnaire data, we constructed an Eastern European diet score (EEDS) from nine food groups which can be considered as traditional in this region. The relationship between categorical (low, moderate, high) and continuous (range 0–18) EEDS and mortality was estimated with Cox-regression.

**Results:**

From 18,852 eligible participants, 2234 died during follow-up. In multivariable adjusted models, participants with high adherence to the traditional Eastern European diet had significantly higher risk of all-cause (HR 1.23; 95% CI 1.08–1.42) and CVD (1.34; 1.08–1.66) deaths compared to those with low adherence. The association with cancer mortality was only significant in Poland (high vs. low EEDS: 1.41; 1.00–1.98). From the specific EEDS components, high consumption of lard was significantly positively related to all three mortality outcomes, while preserved fruit and vegetable consumption showed consistent inverse associations.

**Conclusion:**

Our results suggest that traditional eating habits may contribute to the poor health status, particularly the high CVD mortality rates, of populations in Eastern Europe. Adequate public health nutritional interventions in this region are essential.

**Electronic supplementary material:**

The online version of this article (10.1007/s00394-020-02319-9) contains supplementary material, which is available to authorized users.

## Introduction

Thirty years after the fall of the Iron Curtain, a large health gap persists between Eastern and Western Europe. In 2015, male and female life expectancy at birth in Eastern European countries was on average 8.4 and 4.8 years lower compared to the West, respectively; a difference which has only been marginally reduced over the last two decades [1].

In terms of cause-specific mortality, the East–West divide is particularly striking for cardiovascular disease (CVD), with Russia and some other former Soviet Union countries experiencing mortality rates over three times higher than the Western European average [1]. Although the overall differences for cancer mortality are smaller, countries such as Hungary and Slovakia have some of the highest rates not just in Europe but globally as well [2, 3].

Previous research have explored the potential explanations for the East–West health gap and important social and lifestyle risk factors are identified [4, 5]. Among the lifestyle factors, alcohol and smoking have received the most attention and their role in the poor health of Eastern Europeans is supported by strong evidence [6, 7]. Although unhealthy diet has also been studied, given its complexity and difficulties with its measurement, the number of relevant studies is smaller and the available evidence is considerably weaker. Nevertheless, among the specific dietary factors, inadequate fruit intake and high animal fat consumption are suggested as potential contributors to the high mortality rates of Eastern European populations [8, 9].

Traditional dietary habits can be particularly important to help explain regional differences in health. These diets are characteristic to a specific country or region, and usually consist of foods that have been consumed in that area for an extended period of time [10]. Furthermore, despite the globalisation of food industry and widespread consumption of the Western-style diet, many people still prefer traditional flavours over new dietary trends. In fact, the popularity of traditional food products has been on the rise in Europe, a trend recently further fuelled by extensive interest from industry and marketing [11, 12].

The main aim of this analysis was to examine the relationship of the traditional Eastern European dietary pattern with CVD, cancer and overall mortality rates using prospective data from the Health, Alcohol and Psychosocial factors in Eastern Europe (HAPIEE) study.

## Methods

### Study sample

The HAPIEE study is one of the largest and most recent prospective cohorts with available dietary data in Eastern European countries. The details, including the overall rationale for the study and the methods for recruitment and data collection, have been described previously [13]. Participants were recruited from the general population in Novosibirsk (Russia), Krakow (Poland) and six middle-sized towns in the Czech Republic. The baseline sample included 29,845 men and women with an age range of 45 and 70 years (overall response rate 59%). From the full sample, we included 18,852 individuals in the current analysis. The sample selection process and reasons for exclusion are shown in Figure S1 in Online Resource 1.

All participants signed informed consent. The study protocols were approved by ethical committees at University College London, UK and at each participating centre.

### Data collection and follow-up

Baseline data collection took place between 2002 and 2005. Participants completed a comprehensive questionnaire, provided a blood sample and underwent a brief medical examination. Dietary assessment was carried out using a 9-point scale semi-quantitative food frequency questionnaire (FFQ) with 136, 148 and 147 food and drink items in the Czech Republic, Poland and Russia, respectively [14]. The relative validity of the FFQ data regarding fruit, vegetable and micronutrient intakes was assessed by estimating correlations with concentration biomarkers in a random sub-sample of study subjects. The correlations were found to be similar to other such validation studies and were considered adequate [15].

Local and regional death registers in Krakow and Novosibirsk and national death register in the Czech Republic were used to identify deaths in the study sample. In Poland and the Czech Republic, individuals were followed up for mortality until August and December 2017, respectively. In Russia, death registration data was not fully available between 2011 and 2015 for administrative reasons, therefore participants with no information on their vital status after 2011 were censored in December 2010 (*n* = 1697). For individuals who participated in subsequent data collection between 2015 and 2017, or date and cause of death was ascertained from other sources (i.e.: the Research Institute`s myocardial infarction and stroke registers originally established for the WHO MONICA project), the date of death or last contact was used as the end date of follow-up. Deaths from CVD (ICD-10: I00-I99), cancer (C00-D48) and non-CVD-non-cancer causes (all other ICD codes) were determined using the 10^th^ revision of the International Classification of Diseases.

### Identifying traditional dietary habits in Eastern Europe

In order to identify the traditional Eastern European diet, information on eating habits of rural communities in the 1950s and early 1960s was reviewed across the region. The relevant information sources included historical dietary surveys, ethnographic studies, secondary literature sources and consultations with local experts. The full list of literature sources used for this purpose is shown in Online Resource 2.

Although there are a number of proposed general definitions for traditional food products, the terminology is not consistent and their application in a specific geographical context is problematic [10, 16]. On the other hand, eating habits of rural communities have often been used to characterise traditional dietary patterns of a specific country or wider geographical region. For example, the Mediterranean diet is often described as the “traditional” diet of populations in the Mediterranean region [17]. Its composition is based on dietary data collected in predominantly rural areas in various Mediterranean countries (Italy, Greece, former Yugoslavia) during the late 1950s/early 1960s as part of the Seven Countries Study [18].

Beyond the analogy with Mediterranean diet, there are further reasons why focusing on the rural diet in the 1950s/early 1960s is useful when trying to identify traditional diets in Eastern Europe. Before the mid-1960s, the lifestyle and eating habits of the rural population in most East European countries were still in line with those of earlier decades or centuries. Most of the consumed foods were produced locally and the food preparation and preservation methods did not include modern cooking techniques and contemporary household items [19]. The eating habits of rural communities changed considerably from the mid-1960s for several reasons. Firstly, the use of new, modern household devices, including electric/gas cookers and fridges, slowly became commonplace, impacting on how meals were prepared and what ingredients could be used on a daily basis. Secondly, during the 1960s and 70s, village shops grew in number and size, and purchasing cooking ingredients and ready-to-eat food items (such as flour, bread, meat products, etc.) gradually became the norm [20]. This latter shift from self-sufficiency to dependence on shopping was partly due to convenience, but also the result of collectivisation which made it compulsory for farmers to hand in part of their products (grains, meat, etc.) to local collective farms, leaving them with less than what they and their family needed to survive.

### Construction of the Eastern European Diet Score

Using the above described methodology, nine food groups were identified which were regularly consumed by rural communities in the 1950s/early 1960s in several Eastern European countries. These were (1) bread and grain products, (2) potato, (3) legumes, (4) storable vegetables, (5) preserved fruits and vegetables, (6) dairy products and egg, (7) poultry, (8) processed meat products and (9) lard for cooking. Specific food items from these food groups, relevant to the three HAPIEE countries, and listed in the FFQs are shown in table S1 in Online Resource 1.

The Eastern European diet score (EEDS) was constructed by using consumption data on these nine food groups in the following way (Table 1). First, energy adjusted intakes were calculated with the energy–density method for all components except lard. Subsequently, study participants were assigned 0, 1 or 2 points according to the tertiles of these energy adjusted intakes, based on the pooled distributions across the three countries, with individuals whose intake for a particular food group was in the lowest tertile receiving 0 points and those in the highest tertile receiving 2 points. This scoring system is similar to the relative Mediterranean diet score (rMDS) developed by Buckland et al. [21], which version is also supported by a recent review of MDS methodologies [22]. Regarding lard intake, information was collected not just in the FFQ but also with a separate question that asked what type of fat/oil the participant used most often for cooking. Therefore, for this component, the scoring was different: those who did not cook with lard or consumed it in any other way (i.e.: on bread) received 0 points; those who indicated in the FFQ that they consumed it once a week or less often received 1 point; finally those who reported to regularly cook with lard or consume it more than once a week received 2 points. The EEDS was calculated by adding up the individual component scores, giving an overall range between 0 and 18 points. Similar to the categorisation of the rMDS [21], individuals with EEDS 0–6, 7–10 and 11–18 were considered as having low, moderate or high adherence to the traditional Eastern European diet, respectively.

**Table 1:** Intake values of traditional foods and scoring system of the Eastern European Diet Score (EEDS)

EEDS components	Energy-adjusted^a^ intake (g/day/1000 kcal) percentiles			EEDS component score^b^		
	33.3rd	50th	66.6th	0 point	1 point	2 points
Bread and other grain products	15.0	26.3	40.0	Lowest tertile	Middle tertile	Highest tertile
Potato	33.7	41.5	51.6	Lowest tertile	Middle tertile	Highest tertile
Legumes	3.4	5.4	7.6	Lowest tertile	Middle tertile	Highest tertile
Preserved fruits and vegetables	5.0	7.4	10.6	Lowest tertile	Middle tertile	Highest tertile
Storable vegetables	44.7	60.0	76.0	Lowest tertile	Middle tertile	Highest tertile
Dairy products and egg	19.4	25.2	31.7	Lowest tertile	Middle tertile	Highest tertile
Poultry meat	10.4	17.4	22.4	Lowest tertile	Middle tertile	Highest tertile
Processed meat products	17.0	23.6	30.9	Lowest tertile	Middle tertile	Highest tertile
Lard (%)	n.a	n.a	n.a	Not used for cooking and not consumed it in any other form (66.8)	Not used for cooking but consumed once a week or less (23.0)	Used for cooking and/or consumed more than once a week (10.3)

^a^Mean energy intake of the sample: 2264.3 kcal/day

^b^Lowest, middle and highest tertile refer to energy-adjusted intakes of the specific food groups

### Statistical analysis

The association between EEDS and mortality was assessed with Cox-regression models using calendar time as the underlying time variable and time to death as outcome. EEDS was treated as a categorical variable with three groups (0–6; 7–10; 11–18) using the lowest as reference, and also as a continuous variable examining the risk of mortality per 2-point increase in the score. Proportionality assumptions were tested with Schoenfeld residuals. Since there was no interaction between EEDS and country-cohort or between EEDS and sex, the associations with mortality were estimated in the pooled sample. Nonetheless, cohort-specific results are also presented.

In addition to the overall EEDS, the relationship between the individual EEDS components and mortality outcomes were also assessed. As participants were categorised into three groups for all nine components (0, 1 or 2), we calculated the hazard ratios (HRs) per 1-point increase in the component score.

We applied two multivariable adjusted models for all examined associations. In model 1, the HRs were adjusted for age, sex, country-cohort and energy intake (kcal/day). In model 2, we further adjusted the associations for education (primary or less; vocational, secondary; university), level of deprivation (assessed based on reported difficulties in purchasing clothes, food, or paying the bills—low; moderate; high), marital status (living alone; living with partner), alcohol consumption (abstainers; moderate drinkers: ≤ 15 g/day for women, ≤ 30 g/day for men; heavy drinkers: > 15 g/day for women, > 30 g /day for men), smoking (never smoker; ex-smoker; regular smoker), leisure time physical activity (sport and housework activities expressed in METh/day tertiles—low; moderate; high), fresh fruit intake (g/day) and fish intake (g/day). The non-dietary variables were selected based on previous literature, while fresh fruit and fish intake were added in order to take into account eating habits not captured by the EEDS.

All statistical analyses were carried out using the 15.1 version of the statistical software STATA (StataCorp, Texas, USA).

## Results

Table 2 shows the proportions of participants with high adherence to the traditional Eastern European diet across the covariate categories. Overall, 20% of the sample scored 11 points or more, and this proportion was considerably higher in the Polish cohort compared to Russians and Czechs. As expected, older people were more likely to adhere closely to the traditional pattern, and we also found that a larger proportion of females compared to males scored high. Regarding socio-economic and lifestyle characteristics, individuals with less than primary education, and those who were married, drank no alcohol, did not smoke, did more physical exercise, consumed less energy, and ate more fruits and fish were more likely to score higher in the EEDS.

**Table 2:** Characteristics of the sample by adherence to the traditional Eastern European dietary pattern

Covariate	Subgroup	Adherence to the traditional Eastern European dietary pattern			
		Low (EEDS: 0–6)	Moderate (EEDS: 7–10)	High (EEDS: 11–18)	*p* value^a^
		*n* = 4018	*n* = 11,087	*n* = 3747	
	Full sample (%)	21.3	58.8	19.9	
Country cohort	Czech Republic (%)	22.8	58.7	18.6	< 0.001
	Russia (%)	27.5	60.9	11.7	
	Poland (%)	13.5	56.9	29.6	
Sex	Females (%)	20.5	57.7	21.8	< 0.001
	Males (%)	22.3	60.1	17.7	
Age (years)	45–49 (%)	25.1	60.0	14.9	< 0.001
	50–54 (%)	24.0	58.7	17.3	
	55–59 (%)	21.3	59.1	19.7	
	60–64 (%)	18.2	57.3	24.5	
	65–69 (%)	16.6	58.9	24.5	
Energy intake	Low (< 2000 kcal/day) (%)	16.8	56.5	26.7	< 0.001
	Moderate (2000-2500 kcal/day) (%)	19.2	60.5	20.3	
	High (> 2500 kcal/day) (%)	28.7	60.2	11.1	
Marital status	Married (%)	20.6	59.0	20.4	< 0.001
	Single (%)	23.6	58.1	18.3	
Education	Primary or less (%)	19.2	56.7	24.1	< 0.001
	Vocational (%)	22.0	59.3	18.7	
	Secondary (%)	21.8	58.1	20.1	
	University (%)	20.7	60.2	19.1	
Level of deprivation	Low (%)	20.7	58.8	20.6	0.126
	Moderate (%)	21.6	59.3	19.1	
	High (%)	22.0	58.4	19.6	
Alcohol intake	Abstainer (%)	18.2	57.5	24.4	< 0.001
	Moderate intake (%)	21.4	59.0	19.6	
	High intake (%)	27.0	60.1	12.9	
Smoking	Never smoker (%)	20.7	59.0	20.3	0.002
	Ex-smoker (%)	20.2	59.3	20.5	
	Current smoker (%)	23.1	58.2	18.8	
Leisure-time physical activity	Low (%)	22.6	59.5	17.9	< 0.001
	Moderate (%)	21.8	59.0	19.2	
	High (%)	19.9	58.2	22.0	
Fresh fruit intake tertiles	Low (%)	24.3	59.1	16.6	< 0.001
	Moderate (%)	18.4	59.5	22.2	
	High (%)	21.2	57.9	20.8	
Fish intake tertiles	Low (%)	26.0	57.3	16.7	< 0.001
	Moderate (%)	19.6	59.2	21.3	
	High (%)	18.3	60.0	21.8	

^a^*p* values are calculated with Chi-square test

Table 3 shows the basic (model 1) and multivariable adjusted (model 2) HRs of all-cause, CVD, cancer and other cause (non-CVD-non-cancer) mortality across the three EEDS categories in the pooled sample, as well as in the three country-cohorts separately. The HRs of mortality per 2-point increase in the EEDS are also presented.

**Table 3:** Association between EEDS and all-cause and cause-specific mortality in the pooled and country-specific samples

Outcome	Subsample	*n*	Deaths/1000 person-years	Model	Adherence to the traditional Eastern European dietary pattern					Per 2-pt increase in the EEDS score		Interaction *p* value^a^
					Low (EEDS: 0–6)	Moderate (EEDS: 7–10)		High (EEDS: 11–18)				
					HR	HR	(95%CI)	HR	(95%CI)	HR	(95%CI)	
All-cause	Pooled	18,852	2234/229.1	Model 1	1.00 (ref.)	1.05	(0.94–1.18)	1.11	(0.97–1.27)	1.04	(1.00–1.08)	0.354
				Model 2	1.00 (ref.)	1.13	(1.01–1.26)	1.23	(1.08–1.42)	1.07	(1.03–1.11)	0.509
	Czech Rep	6,100	840/82.6	Model 1	1.00 (ref.)	0.93	(0.77–1.12)	1.06	(0.87–1.31)	1.03	(0.98–1.09)	
				Model 2	1.00 (ref.)	0.98	(0.81–1.18)	1.19	(0.96–1.46)	1.06	(1.01–1.12)	
	Russia	6,474	641/64.2	Model 1	1.00 (ref.)	0.96	(0.79–1.16)	0.97	(0.76–1.25)	0.98	(0.91–1.05)	
				Model 2	1.00 (ref.)	1.11	(0.92–1.35)	1.20	(0.93–1.54)	1.04	(0.98–1.12)	
	Poland	6,278	753/82.2	Model 1	1.00 (ref.)	1.15	(0.95–1.40)	1.27	(1.01–1.59)	1.07	(1.01–1.13)	
				Model 2	1.00 (ref.)	1.15	(0.95–1.41)	1.31	(1.04–1.65)	1.08	(1.02–1.15)	
CVD	Pooled	18,852	883/229.1	Model 1	1.00 (ref.)	1.05	(0.88–1.25)	1.17	(0.95–1.45)	1.01	(0.96–1.07)	0.587
				Model 2	1.00 (ref.)	1.15	(0.96–1.38)	1.34	(1.08–1.66)	1.05	(0.99–1.11)	0.586
	Czech Rep	6,100	301/82.6	Model 1	1.00 (ref.)	1.06	(0.77–1.47)	1.19	(0.83–1.70)	1.05	(0.95–1.15)	
				Model 2	1.00 (ref.)	1.15	(0.83–1.60)	1.39	(0.97–2.00)	1.09	(1.00–1.20)	
	Russia	6,474	324/64.2	Model 1	1.00 (ref.)	1.05	(0.80–1.38)	0.87	(0.60–1.26)	0.92	(0.84–1.02)	
				Model 2	1.00 (ref.)	1.26	(0.95–1.66)	1.10	(0.75–1.60)	1.00	(0.91–1.10)	
	Poland	6,278	258/82.2	Model 1	1.00 (ref.)	0.96	(0.69–1.33)	1.19	(0.82–1.73)	1.03	(0.93–1.14)	
				Model 2	1.00 (ref.)	0.95	(0.68–1.32)	1.21	(0.83–1.77)	1.04	(0.94–1.15)	
Cancer	Pooled	18,852	860/229.1	Model 1	1.00 (ref.)	0.96	(0.80–1.15)	1.08	(0.87–1.33)	1.06	(1.00–1.12)	0.165
				Model 2	1.00 (ref.)	1.01	(0.84–1.21)	1.16	(0.94–1.44)	1.08	(1.02–1.15)	0.187
	Czech Rep	6,100	353/82.6	Model 1	1.00 (ref.)	0.81	(0.61–1.06)	1.01	(0.75–1.38)	1.02	(0.94–1.12)	
				Model 2	1.00 (ref.)	0.82	(0.62–1.09)	1.09	(0.80–1.49)	1.05	(0.96–1.14)	
	Russia	6,474	164/64.2	Model 1	1.00 (ref.)	0.85	(0.59–1.23)	0.95	(0.59–1.54)	1.01	(0.89–1.16)	
				Model 2	1.00 (ref.)	0.92	(0.63–1.34)	1.07	(0.66–1.74)	1.05	(0.92–1.21)	
	Poland	6,278	343/82.2	Model 1	1.00 (ref.)	1.17	(0.87–1.56)	1.36	(0.97–1.90)	1.12	(1.02–1.22)	
				Model 2	1.00 (ref.)	1.17	(0.88–1.57)	1.41	(1.00–1.98)	1.14	(1.04–1.24)	
Other (non-cvd-non-cancer)	Pooled	18,852	491/229.1	Model 1	1.00 (ref.)	1.23	(0.97–1.57)	1.04	(0.77–1.41)	1.05	(0.97–1.14)	0.247
				Model 2	1.00 (ref.)	1.32	(1.04–1.68)	1.16	(0.85–1.57)	1.09	(1.00–1.17)	0.229
	Czech Rep	6,100	186/82.6	Model 1	1.00 (ref.)	1.00	(0.68–1.47)	0.99	(0.63–1.55)	1.01	(0.90–1.14)	
				Model 2	1.00 (ref.)	1.08	(0.73–1.59)	1.11	(0.70–1.75)	1.05	(0.93–1.18)	
	Russia	6,474	153/64.2	Model 1	1.00 (ref.)	0.90	(0.61–1.32)	1.24	(0.76–2.02)	1.06	(0.92–1.22)	
				Model 2	1.00 (ref.)	1.01	(0.68–1.48)	1.54	(0.94–2.51)	1.13	(0.99–1.30)	
	Poland	6,278	152/82.2	Model 1	1.00 (ref.)	1.50	(0.96–2.34)	1.18	(0.68–2.05)	1.02	(0.89–1.17)	
				Model 2	1.00 (ref.)	1.54	(0.98–2.42)	1.23	(0.70–2.15)	1.04	(0.91–1.19)	

Model 1: adjusted for age, sex, country-cohort and energy intake

Model 2: adjusted for model 1 and education, marital status, level of deprivation, smoking, alcohol intake, leisure-time physical activity, fresh fruit intake and fish intake

^a^Interaction by country for the association between EEDS and mortality outcomes. Calculated using likelihood ratio test

Our results show that, after multivariable adjustment (model 2), participants who scored high in the EEDS had significantly higher risk of all-cause mortality compared to low-scorers in the pooled sample (HR 1.23; 95% CI 1.08–1.42). This positive association was also significant when the risk per 2-point increase in the score was assessed (HR 1.07; 95% CI 1.03–1.11). Similar trends were seen in the individual country-cohorts, although some of them did not reach statistical significance.

Regarding cause-specific mortality, we found a significant positive association for CVD in the pooled categorical analysis (HR in high vs. low EEDS 1.34; 95% CI 1.08–1.66), and for cancer when the risk by 2-point increase was estimated (HR 1.08; 95% CI 1.02–1.15). While the direction of the association with CVD was mostly consistent across country-cohorts, for cancer, it was observed only in the Polish sample but not in the Czech and Russian cohorts. Finally, individuals with moderate adherence to the traditional diet had significantly higher risk of other (non-CVD-non-cancer) causes of death compared to low-scorers, but the association was not significant in the high EEDS group, and this result was not consistent across cohorts. Due to the smaller sample size and multiple testing, cause-specific and country-specific results need to be interpreted with caution, as it will be further explained in the Discussion.

Interestingly, most HRs became stronger after full adjustment in model 2, compared to model 1. This observation is likely due to the fact that higher EEDS was associated with healthier lifestyle, as shown in Table 2, which probably masked the real association between EEDS and mortality (negative confounding).

Table 4 shows the associations between the nine traditional food groups that make up the EEDS and the four mortality outcomes in the pooled sample. The HRs indicate the multivariable adjusted risk of mortality per one point increase in the component scores. The strongest and most consistent associations were found for preserved fruits and vegetables and for lard. Participants who consumed higher amounts of preserved fruits and vegetables had significantly lower risk of death due to all-causes, CVD and cancer. In contrast, those who reported regular consumption of lard had significantly higher risk of death for these three mortality outcomes compared to low- or non-consumers. In addition to these two food groups, we also found significant positive links with CVD mortality for intakes of potato and dairy products and egg, with cancer mortality for bread and other grain products, and with other causes of death for potato. Potato, dairy product and egg and storable vegetable intakes were also found to be significantly positively related to all-cause mortality.

**Table 4:** Association between EEDS components and all-cause and cause-specific mortality in the pooled sample (*n* = 18,852)

Component of the EEDS	Mortality outcomes							
	All-cause mortality		CVD		Cancer		Other cause (non-CVD-non-cancer)	
	HR^a^	(95% CI)	HR^a^	(95% CI)	HR^a^	(95% CI)	HR^a^	(95% CI)
Bread and grain products	1.05	(1.00–1.11)	1.02	(0.94–1.11)	1.12	(1.03–1.22)	0.98	(0.88–1.09)
Potato	1.08	(1.03–1.14)	1.12	(1.03–1.22)	1.02	(0.94–1.11)	1.13	(1.01–1.27)
Legumes	1.03	(0.97–1.08)	1.06	(0.97–1.16)	1.04	(0.95–1.13)	0.95	(0.84–1.06)
Preserved fruits and vegetables	0.90	(0.85–0.95)	0.86	(0.79–0.93)	0.88	(0.81–0.96)	1.03	(0.91–1.15)
Storable vegetables	1.07	(1.01–1.13)	1.05	(0.96–1.16)	1.07	(0.97–1.17)	1.09	(0.96–1.23)
Dairy and egg	1.09	(1.04–1.15)	1.11	(1.02–1.21)	1.06	(0.97–1.15)	1.13	(1.00–1.26)
Poultry meat	0.98	(0.93–1.04)	0.93	(0.86–1.01)	1.05	(0.97–1.15)	0.97	(0.87–1.09)
Processed meat	1.02	(0.97–1.08)	1.01	(0.93–1.10)	1.02	(0.93–1.11)	1.05	(0.93–1.17)
Lard	1.12	(1.05–1.19)	1.11	(1.01–1.22)	1.13	(1.02–1.24)	1.12	(0.98–1.27)

All HRs are adjusted for age, sex, country-cohort, energy intake, education, marital status, level of deprivation, smoking, alcohol intake, leisure-time physical activity, fresh fruit intake and fish intake

^a^HR per 1-point increase in the EEDS component score (0, 1 or 2)

Country-specific results for the individual food groups showed mostly similar trends to the pooled analysis, however, due to the smaller sample size, most associations were statistically not significant (Table S2 in Online Resource 1).

The results remained essentially the same when the associations were further adjusted for BMI or when the specific food groups were adjusted for each other (Tables S3 and S4 in Online Resource 1). Similarly, no major change to the main results were observed if missing covariate data were imputed with multiple random imputation procedure, and when Russian participants who were censored in December 2010 were excluded from the analysis (Tables S5, S6 and S7 in Online Resource 1).

## Discussion

### Summary of main findings

Using a cross-disciplinary approach, in this study we identified an `a priori` dietary pattern which can be characterised as traditional in Eastern European populations, and assessed its relationship with all-cause and cause-specific mortality outcomes in Czech, Russian and Polish participants of the HAPIEE study. We found that individuals who adhered closely to the traditional Eastern European dietary pattern had higher risk of deaths from all-causes and from CVD compared to those who did not follow this diet. Cancer mortality rates were also positively associated with this eating pattern in Poland. From the individual components, preserved fruit and vegetable intake was inversely related to the risk of all-cause, CVD and cancer deaths, while regular lard consumption was found to be positively linked with these outcomes.

### Interpretation

Identifying traditional food products and defining the traditional eating pattern of a specific population is a challenging task. However, considering the potential importance of these foods and diets in explaining regional differences in population health, the public health value of such efforts is evident. While we acknowledge the complexities and controversies around using the term “traditional” [23], we also believe that the proposed Eastern European diet score, based on consumption of food products dominant in rural Eastern European communities in the 1950s/early-1960s, is a good indicator of traditional eating habits within the region. In addition to the reasons described in the Methods section, the identified food groups also correspond fairly well with the previously suggested general definitions for “traditional food products” [10, 16].

Participants in our study who closely followed the traditional eating pattern (by, for example, eating relatively large amounts of more than five out of the nine traditional food groups) were at higher risk of death due to all-causes and CVD, as well as cancer in Poland. This indicates that this diet can contribute to the poor health of populations in Eastern European countries. Although high adherence was observed in only 20% of our sample, it is likely that this proportion would have been considerably higher in the 1970s, 80s and 90s when the large health gap between East and West developed, possibly resulting in comparable population attributable risk estimates to smoking and alcohol intake. Previous studies suggested that low fruit and high animal fat intake have contributed to the increased CVD mortality rates in Poland, Russia and other Eastern European states [8, 9]. Our study adds to this evidence by emphasising the role of overall dietary patterns in addition to individual food items.

From the specific food groups which were identified as traditional, preserved fruits and vegetables showed strong inverse associations with CVD, cancer and all-cause mortality outcomes. Although the available previous evidence regarding the health effects of these foods is limited [24], our findings support the view that fruits and vegetables even in preserved forms can be protective against chronic diseases, most likely due to their high antioxidant, fibre, potassium and phytochemical content [25]. Additionally, biologically active compounds that are produced during fermentation, for example in sauerkraut, may also contribute to this beneficial effect [26]. In fact, including this (healthful) food group in the EEDS attenuates the increased relative risk associated with the overall score, and without it, the examined associations were stronger (Table S8 in Online Resource 1). However, as preserved fruits and vegetables are part of the traditional diet we felt it would be inappropriate to omit this component when the adherence to the overall dietary pattern is assessed.

On the other hand, regular consumption of lard, primarily used for cooking, significantly increased the risk of death from both CVD and cancer. Only few epidemiological studies examined the consumption of lard in relation to any health outcomes to date. Nevertheless, considering its high saturated fatty acid content, the adverse health effect is not surprising and consistent with previous evidence [27, 28]. In fact, this food item would be an ideal target for public health campaigns. Modifying cooking practices by encouraging the use of unsaturated fatty-acid rich olive oil or vegetable oil instead of lard could potentially have a large impact on the health of communities where its usage is still dominant.

In addition to the consistent results regarding lard and preserved fruit and vegetables, significant positive associations with specific outcomes were also found for intakes of bread, potato, storable vegetables and dairy products. While we need to be cautious when interpreting these results due to the issue of multiple testing [29], they also merit further consideration. Potatoes and vegetables are often consumed with added salt, which, in turn, can have a considerable impact on CVD risk. Furthermore, these food products were strongly related to indicators of socio-economic position (SEP), and although we adjusted the results for education and level of deprivation, residual confounding by SEP may still play a role. Dairy products often have high fat content in Eastern European countries (i.e.: soured cream), which could be responsible for the increased CVD risk. Finally, our results regarding the positive link between bread intake and cancer mortality, which was particularly prominent in Poland, needs further research, ideally examining the risk in relation to specific types of cancer. This would be especially important as a previous study showed that bread is the main source of acrylamide exposure in the Polish population [30].

### Limitations and strengths

Our study has a number of limitations which need to be considered when interpreting the results.

First of all, Eastern Europe is a diverse region (similar to the Mediterranean territory), therefore it is inevitable that important differences exist in the traditional eating habits between countries and between specific sub-regions within this area. In the current analysis we particularly focused on the Czech Republic, Russia and Poland, the three countries of the HAPIEE study, and it is possible that the specific foods which can be considered traditional may differ in other Eastern European states. However, the nine traditional food groups were identified using historical data from a wide range of countries, so while there may be differences in specific foods, these larger food groups are probably characteristic of most countries within the region.

Secondly, although participants of the HAPIEE study were recruited randomly from the general population, due to the moderate response rate and the fact that the sampling frame included only urban populations, they are not entirely representative to their source countries. This limitation can particularly influence the descriptive findings, providing, for example, lower estimates for the proportion of participants with regular lard intake or high adherence to the traditional eating pattern than if rural samples had also been included. Nonetheless, the internal validity of our findings regarding the associations between EEDS and mortality outcomes are probably less affected by the limited representativeness of the sample.

Dietary intakes are notoriously difficult to measure precisely in any population, and the FFQ has well-known limitations [31]. However, we used relative consumption data (tertiles) to calculate component scores for eight out of the nine traditional food groups, as opposed to absolute intakes, and we also adjusted the values for energy intake. These were two important steps to reduce the impact of measurement bias [32]. Nevertheless, the misclassification, if random, is likely to lead to underestimation of the underlying relative risks.

Our study has important strengths as well. The multi-disciplinary approach which involved epidemiologists, public health specialists, as well as sociologists, economists and historians from several relevant countries ensured that the characterisation of traditional Eastern European foods was as comprehensive as possible. It is also a good example of a truly cross-disciplinary research within the field of nutritional epidemiology and public health, which is still rare despite widespread calls for such approaches. In fact, this work may be viewed as a proof-of-concept, and the methodology could be potentially applied to traditional diets in other geographical contexts as well.

A further advantage of this study is that the construction of the EEDS utilised the accumulated evidence from similar procedures applied to the Mediterranean diet. Additionally, the large sample size of the HAPIEE dataset, as well as the fact that the associations could be tested in three specific countries, provided further strengths to our analysis in terms of statistical power and the ability to evaluate cross-cultural consistency of the results.

## Conclusion

This is the first study which attempted to identify traditional dietary habits in Eastern European populations and assessed the relationship between this eating pattern and mortality outcomes. The results suggest a positive association of traditional Eastern European diet with increased mortality. Although further research in this field is clearly needed, the results provide evidence for nutritional public health interventions in the region.

## Electronic supplementary material

Below is the link to the electronic supplementary material.

Supplementary file1 (PDF 716 kb)

Supplementary file2 (PDF 609 kb)
