# Supplementary material for: Traditional Eastern European diet and mortality: prospective evidence from the HAPIEE study
Source: Eur J Nutr. 2020 Jul 1;60(2):1091–100. doi: 10.1007/s00394-020-02319-9 (PMC7900332; doi:10.1007/s00394-020-02319-9)
Supplement: Supplementary file 1 — Supplementary file1 (PDF 716 kb) [file 394_2020_2319_MOESM1_ESM.pdf]

ONLINE RESOURCE 1: Additional figures and tables

Manuscript title: Traditional Eastern European diet and mortality: prospective evidence from the HAPIEE study

Journal Name: European Journal of Nutrition

Authors: Denes Stefler, Daniel Brett, Eszter Sarkadi-Nagy, Ewa Kopczynska, Stefan Detchev, Aniko Bati, Mircea Scrob, Diane Koenker, Bojan Aleksov, Elodie Douarin, Galina Simonova, Sofia Malyutina, Ruzena Kubinova, Andrzej Pajak, Milagros Ruiz, Anne Peasey, Hynek Pikhart, Martin Bobak

Corresponding author: Denes Stefler

Department of Epidemiology and Public Health, University College London

Email: denes.stefler@ucl.ac.uk

<u>Figure S1.</u> Sample selection process and reasons for exclusion

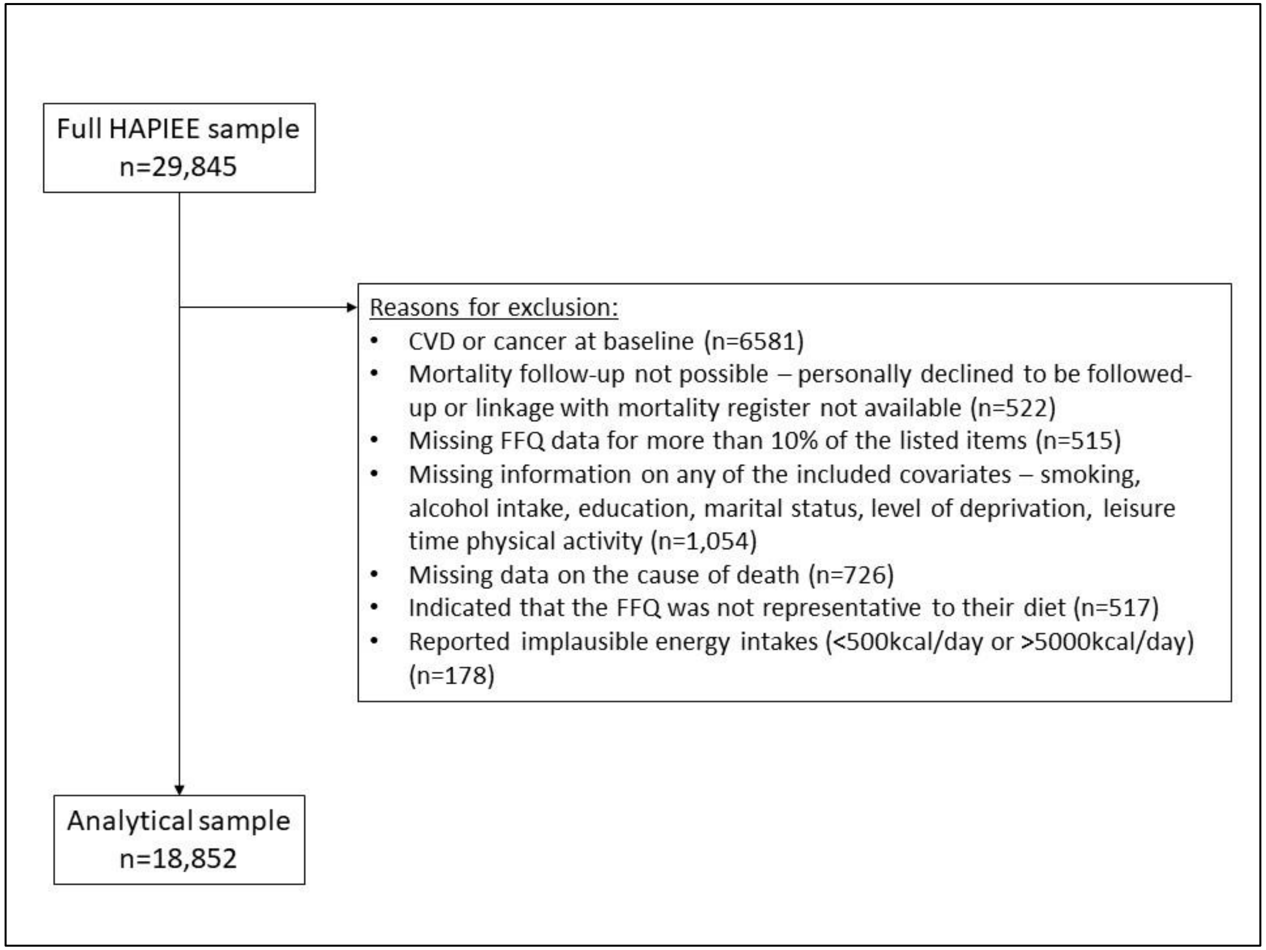

Table S1. Traditional Eastern European food groups and corresponding FFQ items in the HAPIEE study

"Traditional" food group	FFQ item
Bread, grains	Brown/rye bread, dumplings, groats
Potato	Mashed, boiled or baked potatoes, potato dumplings
Legumes	Beans, lentils, dried peas, green peas
Storable vegetables	Cabbage, beetroot, swede, carrot, radish, celeriac, parsnip, onion, vegetable soup (usually made of root vegetables), borsch
Preserved fruit and vegetables	Sauerkraut, pickled gherkin, jam
Dairy products and egg	Soured cream, cheese, cottage cheese, milk, egg
Poultry meat	Poultry meat
Processed meat	Frankfurter sausage, kabana, cervelat, salami, ham, bacon, pate, minced beef
Lard	Lard on bread; lard in food; lard used for cooking

Table S2. Association between EEDS components and all-cause and cause-specific mortality in the country-specific samples

Country-cohort	Component of the EEDS	All-cause mortality		CVD		Cancer		Other cause (non-CVD-non-cancer)	
		HR[a]	(95%CI)	HR[a]	(95%CI)	HR[a]	(95%CI)	HR[a]	(95%CI)
Czech Republic	Bread and grain products	**1.07**	(0.98-1.16)	**1.11**	(0.96-1.27)	**1.11**	(0.97-1.26)	**0.95**	(0.80-1.13)
	Potato	**1.06**	(0.98-1.16)	**1.11**	(0.96-1.28)	**1.01**	(0.89-1.15)	**1.10**	(0.92-1.32)
	Legumes	**1.02**	(0.94-1.12)	**1.17**	(1.01-1.35)	**0.94**	(0.82-1.08)	**0.98**	(0.82-1.17)
	Preserved fruits and vegetables	**0.90**	(0.82-0.98)	**0.89**	(0.77-1.02)	**0.91**	(0.80-1.04)	**0.89**	(0.74-1.07)
	Storable vegetables	**1.04**	(0.95-1.13)	**1.04**	(0.90-1.21)	**1.07**	(0.94-1.23)	**0.97**	(0.81-1.17)
	Dairy and egg	**1.04**	(0.95-1.13)	**1.06**	(0.92-1.22)	**0.99**	(0.87-1.13)	**1.10**	(0.92-1.32)
	Poultry meat	**0.98**	(0.90-1.06)	**0.93**	(0.80-1.07)	**1.06**	(0.93-1.21)	**0.92**	(0.77-1.10)
	Processed meat	**1.11**	(1.02-1.22)	**1.11**	(0.96-1.29)	**1.07**	(0.94-1.23)	**1.21**	(1.00-1.46)
	Lard	**1.11**	(1.01-1.22)	**1.07**	(0.91-1.25)	**1.09**	(0.94-1.26)	**1.22**	(1.00-1.49)
Russia	Bread and grain products	**1.04**	(0.94-1.15)	**1.07**	(0.93-1.23)	**1.01**	(0.84-1.23)	**1.02**	(0.83-1.25)
	Potato	**1.12**	(1.01-1.23)	**1.17**	(1.01-1.35)	**0.99**	(0.82-1.21)	**1.18**	(0.96-1.44)
	Legumes	**0.99**	(0.90-1.09)	**0.87**	(0.76-1.00)	**1.21**	(1.00-1.47)	**1.02**	(0.83-1.24)
	Preserved fruits and vegetables	**0.95**	(0.86-1.05)	**0.90**	(0.78-1.03)	**0.90**	(0.74-1.09)	**1.12**	(0.92-1.37)
	Storable vegetables	**0.96**	(0.87-1.07)	**0.93**	(0.80-1.08)	**0.99**	(0.80-1.22)	**1.00**	(0.81-1.25)
	Dairy and egg	**1.06**	(0.96-1.17)	**1.10**	(0.97-1.26)	**1.01**	(0.83-1.22)	**1.02**	(0.84-1.24)
	Poultry meat	**1.06**	(0.96-1.17)	**0.96**	(0.84-1.10)	**1.13**	(0.93-1.37)	**1.18**	(0.97-1.44)
	Processed meat	**0.99**	(0.89-1.09)	**0.96**	(0.84-1.10)	**1.03**	(0.85-1.25)	**0.99**	(0.81-1.20)
	Lard	**1.04**	(0.92-1.18)	**1.09**	(0.92-1.29)	**0.93**	(0.70-1.23)	**1.04**	(0.81-1.35)
Poland	Bread and grain products	**1.07**	(0.97-1.17)	**0.96**	(0.82-1.13)	**1.16**	(1.01-1.33)	**1.05**	(0.86-1.29)
	Potato	**1.05**	(0.96-1.16)	**1.08**	(0.91-1.28)	**1.12**	(0.98-1.29)	**0.86**	(0.69-1.06)
	Legumes	**1.05**	(0.96-1.15)	**1.07**	(0.92-1.25)	**1.06**	(0.93-1.22)	**1.00**	(0.82-1.23)
	Preserved fruits and vegetables	**0.92**	(0.85-1.01)	**0.84**	(0.72-0.98)	**0.94**	(0.83-1.07)	**1.05**	(0.86-1.28)
	Storable vegetables	**1.07**	(0.97-1.17)	**1.13**	(0.97-1.32)	**1.09**	(0.95-1.25)	**0.91**	(0.74-1.12)
	Dairy and egg	**1.12**	(1.02-1.22)	**1.03**	(0.89-1.20)	**1.10**	(0.96-1.25)	**1.31**	(1.08-1.60)
	Poultry meat	**0.93**	(0.85-1.01)	**0.91**	(0.78-1.05)	**0.96**	(0.84-1.10)	**0.90**	(0.74-1.09)
	Processed meat	**1.05**	(0.96-1.15)	**1.07**	(0.92-1.25)	**1.01**	(0.89-1.16)	**1.11**	(0.91-1.36)
	Lard	**1.19**	(1.07-1.31)	**1.20**	(1.01-1.44)	**1.25**	(1.08-1.45)	**1.03**	(0.81-1.30)

Mortality outcomes: All-cause mortality, CVD, Cancer, Other cause (non-CVD-non-cancer)

All HRs are adjusted for age, sex, energy intake, education, marital status, level of deprivation, smoking, alcohol intake, leisure-time physical activity, fresh fruit intake and fish intake

[a] HR per 1-point increase in the EEDS component score (0, 1 or 2)

<u>Table S3.</u> Association between EEDS and all-cause and cause-specific mortality in the pooled and country-specific samples **after further adjustment for BMI**

Outcome	Subsample	n	deaths/1000 person-years	Adherence to the traditional Eastern European dietary pattern: Low (EEDS: 0-6)	Moderate (EEDS: 7-10)		High (EEDS: 11-18)		Per 2-pt increase in the EEDS score	
				HR	HR	(95%CI)	HR	(95%CI)	HR	(95%CI)
All-cause	Pooled	18,828	2,227/228.8	**1.00 (ref.)**	**1.12**	(1.01-1.26)	**1.23**	(1.07-1.41)	**1.07**	(1.03-1.11)
	Czech Rep.	6,084	837/82.4	**1.00 (ref.)**	**0.96**	(0.80-1.16)	**1.16**	(0.94-1.43)	**1.06**	(1.00-1.11)
	Russia	6,474	641/64.2	**1.00 (ref.)**	**1.11**	(0.92-1.35)	**1.20**	(0.93-1.54)	**1.04**	(0.98-1.12)
	Poland	6,270	749/82.2	**1.00 (ref.)**	**1.17**	(0.96-1.43)	**1.32**	(1.05-1.67)	**1.08**	(1.02-1.15)
CVD	Pooled	18,828	882/228.8	**1.00 (ref.)**	**1.15**	(0.95-1.35)	**1.31**	(1.05-1.62)	**1.04**	(0.99-1.11)
	Czech Rep.	6,084	300/82.4	**1.00 (ref.)**	**1.10**	(0.80-1.53)	**1.30**	(0.91-1.88)	**1.08**	(0.98-1.19)
	Russia	6,474	324/64.2	**1.00 (ref.)**	**1.26**	(0.96-1.66)	**1.10**	(0.75-1.60)	**1.00**	(0.91-1.10)
	Poland	6,270	258/82.2	**1.00 (ref.)**	**0.95**	(0.68-1.32)	**1.19**	(0.82-1.74)	**1.03**	(0.93-1.15)
Cancer	Pooled	18,828	857/228.8	**1.00 (ref.)**	**1.00**	(0.83-1.20)	**1.15**	(0.92-1.42)	**1.08**	(1.01-1.14)
	Czech Rep.	6,084	352/82.4	**1.00 (ref.)**	**0.80**	(0.61-1.06)	**1.06**	(0.77-1.45)	**1.04**	(0.95-1.14)
	Russia	6,474	164/64.2	**1.00 (ref.)**	**0.92**	(0.63-1.34)	**1.07**	(0.66-1.74)	**1.05**	(0.92-1.21)
	Poland	6,270	341/82.2	**1.00 (ref.)**	**1.17**	(0.87-1.56)	**1.40**	(0.99-1.97)	**1.13**	(1.04-1.24)
Other cause	Pooled	18,828	488/228.8	**1.00 (ref.)**	**1.35**	(1.06-1.72)	**1.21**	(0.89-1.64)	**1.10**	(1.02-1.19)
	Czech Rep.	6,084	185/82.4	**1.00 (ref.)**	**1.11**	(0.74-1.65)	**1.15**	(0.72-1.82)	**1.06**	(0.94-1.19)
	Russia	6,474	153/64.2	**1.00 (ref.)**	**1.01**	(0.69-1.49)	**1.54**	(0.94-2.51)	**1.13**	(0.99-1.30)
	Poland	6,270	150/82.2	**1.00 (ref.)**	**1.69**	(1.06-2.69)	**1.39**	(0.78-2.47)	**1.06**	(0.93-1.22)

All HRs are adjusted for age, sex, country-cohort, energy intake, education, marital status, level of deprivation, smoking, alcohol intake, leisure-time physical activity, fresh fruit intake, fish intake and BMI

<u>Table S4.</u> Association between EEDS components and all-cause and cause-specific mortality in the pooled sample **after adjusting the components for each other and for BMI**

Component of the EEDS	All-cause mortality		CVD		Cancer		Other cause (non-CVD-non-cancer)	
	HR[a]	(95%CI)	HR[a]	(95%CI)	HR[a]	(95%CI)	HR[a]	(95%CI)
Bread and grain products	**1.05**	(1.00-1.11)	**1.02**	(0.95-1.11)	**1.12**	(1.03-1.22)	**0.99**	(0.88-1.11)
Potato	**1.09**	(1.03-1.15)	**1.13**	(1.04-1.23)	**1.02**	(0.94-1.11)	**1.15**	(1.02-1.29)
Legumes	**1.02**	(0.96-1.07)	**1.07**	(0.98-1.17)	**1.02**	(0.93-1.12)	**0.93**	(0.83-1.04)
Preserved fruits and vegetables	**0.90**	(0.85-0.95)	**0.85**	(0.78-0.93)	**0.88**	(0.80-0.96)	**1.03**	(0.92-1.16)
Storable vegetables	**1.08**	(1.02-1.15)	**1.07**	(0.97-1.18)	**1.07**	(0.97-1.18)	**1.09**	(0.96-1.23)
Dairy and egg	**1.11**	(1.05-1.17)	**1.11**	(1.02-1.21)	**1.07**	(0.98-1.17)	**1.13**	(0.99-1.28)
Poultry meat	**0.99**	(0.93-1.04)	**0.93**	(0.86-1.01)	**1.06**	(0.97-1.15)	**0.97**	(0.87-1.09)
Processed meat	**1.02**	(0.97-1.08)	**1.00**	(0.92-1.09)	**1.01**	(0.93-1.11)	**1.08**	(0.96-1.21)
Lard	**1.13**	(1.06-1.20)	**1.12**	(1.01-1.24)	**1.14**	(1.03-1.26)	**1.13**	(0.99-1.29)

Mortality outcomes: All-cause mortality, CVD, Cancer, Other cause (non-CVD-non-cancer)

All HRs are adjusted for age, sex, country-cohort, energy intake, education, marital status, level of deprivation, smoking, alcohol intake, leisure-time physical activity, fresh fruit intake, fish intake, BMI and all other components

[a] HR per 1-point increase in the EEDS component score (0, 1 or 2)

Table S5. Association between EEDS and all-cause and cause-specific mortality in the pooled and country-specific samples **after multiple random imputation[a] of missing covariate data (n=19,821)**

Outcome	Subsample	n	deaths/1000 person-years	Adherence to the traditional Eastern European dietary pattern: Low (EEDS: 0-6)	Moderate (EEDS: 7-10)		High (EEDS: 11-18)		Per 2-pt increase in the EEDS score	
				HR	HR	(95%CI)	HR	(95%CI)	HR	(95%CI)
All-cause	Pooled	19,821	2,418/241.4	**1.00 (ref.)**	**1.10**	(0.99-1.22)	**1.23**	(1.08-1.40)	**1.07**	(1.03-1.11)
	Czech Rep.	6,581	939/88.9	**1.00 (ref.)**	**1.00**	(0.83-1.19)	**1.21**	(0.99-1.48)	**1.07**	(1.01-1.12)
	Russia	6,486	647/64.2	**1.00 (ref.)**	**1.10**	(0.91-1.33)	**1.20**	(0.93-1.55)	**1.05**	(0.98-1.13)
	Poland	6,754	832/88.3	**1.00 (ref.)**	**1.10**	(0.92-1.33)	**1.25**	(1.01-1.56)	**1.07**	(1.01-1.13)
CVD	Pooled	19,821	951/241.4	**1.00 (ref.)**	**1.09**	(0.92-1.30)	**1.28**	(1.04-1.58)	**1.04**	(0.99-1.10)
	Czech Rep.	6,581	332/88.9	**1.00 (ref.)**	**1.17**	(0.86-1.61)	**1.38**	(0.97-1.96)	**1.09**	(0.99-1.19)
	Russia	6,486	324/64.2	**1.00 (ref.)**	**1.24**	(0.94-1.63)	**1.11**	(0.76-1.62)	**1.00**	(0.91-1.10)
	Poland	6,754	295/88.3	**1.00 (ref.)**	**0.89**	(0.65-1.20)	**1.16**	(0.82-1.65)	**1.02**	(0.93-1.13)
Cancer	Pooled	19,821	919/241.4	**1.00 (ref.)**	**0.97**	(0.81-1.15)	**1.17**	(0.95-1.44)	**1.08**	(1.02-1.14)
	Czech Rep.	6,581	388/88.9	**1.00 (ref.)**	**0.80**	(0.62-1.04)	**1.07**	(0.80-1.44)	**1.04**	(0.96-1.13)
	Russia	6,486	165/64.2	**1.00 (ref.)**	**0.93**	(0.64-1.36)	**1.03**	(0.63-1.70)	**1.06**	(0.92-1.21)
	Poland	6,754	366/88.3	**1.00 (ref.)**	**1.17**	(0.88-1.55)	**1.39**	(0.99-1.93)	**1.13**	(1.03-1.23)
Other cause	Pooled	19,821	548/241.4	**1.00 (ref.)**	**1.35**	(1.08-1.70)	**1.21**	(0.90-1.62)	**1.09**	(1.01-1.18)
	Czech Rep.	6,581	219/88.9	**1.00 (ref.)**	**1.19**	(0.81-1.73)	**1.30**	(0.84-1.99)	**1.09**	(0.97-1.21)
	Russia	6,486	158/64.2	**1.00 (ref.)**	**0.99**	(0.68-1.45)	**1.56**	(0.96-2.54)	**1.15**	(1.00-1.32)
	Poland	6,754	171/88.3	**1.00 (ref.)**	**1.39**	(0.93-2.10)	**1.10**	(0.65-2.85)	**1.04**	(0.91-1.18)

All HRs are adjusted for age, sex, country-cohort, energy intake, education, marital status, level of deprivation, smoking, alcohol intake, leisure-time physical activity, fresh fruit intake and fish intake

[a] Multiple chained imputation procedure was used in Stata v 15.1. Ten imputed datasets were created, and in addition to all variables which the associations were adjusted for (listed above), data on all-cause, CVD, cancer and other cause (non-CVD-non-cancer) mortality, as well as BMI were also included in the imputation procedure.

Table S6. Association between EEDS components and all-cause and cause-specific mortality in the pooled sample **after multiple random imputation[a] of missing covariate data (n=19,821)**

Component of the EEDS	Mortality outcomes							
	All-cause mortality		CVD		Cancer		Other cause (non-CVD-non-cancer)	
	HR[b]	(95%CI)	HR[b]	(95%CI)	HR[b]	(95%CI)	HR[b]	(95%CI)
Bread and grain products	**1.05**	(1.00-1.10)	**1.03**	(0.95-1.11)	**1.09**	(1.01-1.18)	**1.00**	(0.90-1.11)
Potato	**1.08**	(1.03-1.14)	**1.12**	(1.03-1.21)	**1.03**	(0.94-1.12)	**1.13**	(1.01-1.26)
Legumes	**1.04**	(0.99-1.10)	**1.07**	(0.98-1.16)	**1.04**	(0.96-1.13)	**0.99**	(0.89-1.10)
Preserved fruits and vegetables	**0.89**	(0.84-0.93)	**0.84**	(0.77-0.92)	**0.88**	(0.81-0.96)	**0.98**	(0.88-1.10)
Storable vegetables	**1.06**	(1.00-1.12)	**1.03**	(0.94-1.12)	**1.09**	(1.00-1.19)	**1.06**	(0.94-1.19)
Dairy and egg	**1.09**	(1.04-1.15)	**1.09**	(1.01-1.19)	**1.07**	(0.98-1.17)	**1.14**	(1.02-1.27)
Poultry meat	**0.97**	(0.93-1.03)	**0.92**	(0.85-1.00)	**1.03**	(0.95-1.11)	**0.99**	(0.89-1.10)
Processed meat	**1.02**	(0.97-1.08)	**1.02**	(0.94-1.10)	**1.02**	(0.94-1.11)	**1.05**	(0.94-1.17)
Lard	**1.13**	(1.07-1.20)	**1.14**	(1.04-1.25)	**1.14**	(1.04-1.25)	**1.10**	(0.97-1.25)

All HRs are adjusted for age, sex, country-cohort, energy intake, education, marital status, level of deprivation, smoking, alcohol intake, leisure-time physical activity, fresh fruit intake and fish intake

[a] Multiple chained imputation procedure was used in Stata v 15.1. Ten imputed datasets were created, and in addition to all variables which the associations were adjusted for (listed above), data on all-cause, CVD, cancer and other cause (non-CVD-non-cancer) mortality, as well as BMI were also included in the imputation procedure

[b] HR per 1-point increase in the EEDS component score (0, 1 or 2)

<u>Table S7.</u> Association between EEDS and all-cause and cause-specific mortality in the pooled and Russian samples **after excluding Russian participants who were censored in December 2010**

Outcome	Subsample	n	deaths/1000 person-years	Adherence to the traditional Eastern European dietary pattern: Low (EEDS: 0-5) HR	Moderate (EEDS: 6-9) HR	Moderate (95%CI)	High (EEDS: 10-16) HR	High (95%CI)	Per 2-pt increase in the EEDS score HR	(95%CI)
All-cause	Pooled	17,155	2,234/218.0	**1.00 (ref.)**	**1.13**	(1.01-1.27)	**1.24**	(1.08-1.42)	**1.07**	(1.03-1.11)
	Russia	4,777	641/53.1	**1.00 (ref.)**	**1.15**	(0.95-1.39)	**1.28**	(0.99-1.65)	**1.07**	(1.00-1.15)
CVD	Pooled	17,155	883/218.0	**1.00 (ref.)**	**1.15**	(0.97-1.38)	**1.35**	(1.08-1.67)	**1.06**	(1.00-1.12)
	Russia	4,777	324/53.1	**1.00 (ref.)**	**1.29**	(0.98-1.70)	**1.17**	(0.80-1.71)	**1.02**	(0.93-1.12)
Cancer	Pooled	17,155	860/218.0	**1.00 (ref.)**	**1.01**	(0.84-1.21)	**1.16**	(0.94-1.44)	**1.08**	(1.02-1.15)
	Russia	4,777	164/53.1	**1.00 (ref.)**	**0.95**	(0.65-1.39)	**1.15**	(0.70-1.87)	**1.08**	(0.94-1.24)
Other cause	Pooled	17,155	491/218.0	**1.00 (ref.)**	**1.33**	(1.05-1.70)	**1.16**	(0.86-1.58)	**1.09**	(1.01-1.18)
	Russia	4,777	153/53.1	**1.00 (ref.)**	**1.05**	(0.71-1.54)	**1.63**	(1.00-2.66)	**1.17**	(1.02-1.34)

All HRs are adjusted for age, sex, country-cohort, energy intake, education, marital status, level of deprivation, smoking, alcohol intake, leisure-time physical activity, fresh fruit intake and fish intake

Table S8. Association between EEDS and all-cause and cause-specific mortality in the pooled and country-specific samples **without the preserved fruit and vegetable EEDS component**

Outcome	Subsample	n	deaths/1000 person-years	Adherence to the traditional Eastern European dietary pattern: Low (EEDS: 0-5)	Moderate (EEDS: 6-9)		High (EEDS: 10-16)		Per 2-pt increase in the EEDS score	
				HR	HR	(95%CI)	HR	(95%CI)	HR	(95%CI)
All-cause	Pooled	18,852	2,234/229.1	**1.00 (ref.)**	**1.20**	(1.07-1.35)	**1.40**	(1.21-1.62)	**1.11**	(1.07-1.15)
	Czech Rep.	6,100	840/82.6	**1.00 (ref.)**	**1.04**	(0.86-1.27)	**1.30**	(1.04-1.62)	**1.06**	(1.01-1.12)
	Russia	6,474	641/64.2	**1.00 (ref.)**	**1.26**	(1.03-1.54)	**1.35**	(1.03-1.77)	**1.04**	(0.98-1.12)
	Poland	6,278	753/82.2	**1.00 (ref.)**	**1.21**	(0.98-1.49)	**1.51**	(1.18-1.92)	**1.08**	(1.02-1.15)
CVD	Pooled	18,852	883/229.1	**1.00 (ref.)**	**1.13**	(0.94-1.35)	**1.39**	(1.11-1.73)	**1.10**	(1.04-1.17)
	Czech Rep.	6,100	301/82.6	**1.00 (ref.)**	**1.12**	(0.80-1.58)	**1.63**	(1.13-2.38)	**1.09**	(1.00-1.20)
	Russia	6,474	324/64.2	**1.00 (ref.)**	**1.22**	(0.92-1.61)	**1.33**	(0.91-1.94)	**1.00**	(0.91-1.10)
	Poland	6,278	258/82.2	**1.00 (ref.)**	**1.07**	(0.75-1.53)	**1.44**	(0.96-2.16)	**1.04**	(0.94-1.15)
Cancer	Pooled	18,852	860/229.1	**1.00 (ref.)**	**1.15**	(0.95-1.40)	**1.45**	(1.15-1.82)	**1.13**	(1.06-1.20)
	Czech Rep.	6,100	353/82.6	**1.00 (ref.)**	**0.85**	(0.64-1.13)	**1.03**	(0.74-1.43)	**1.05**	(0.96-1.14)
	Russia	6,474	164/64.2	**1.00 (ref.)**	**1.33**	(0.89-2.00)	**1.22**	(0.70-2.13)	**1.05**	(0.92-1.21)
	Poland	6,278	343/82.2	**1.00 (ref.)**	**1.29**	(0.95-1.76)	**1.83**	(1.27-2.61)	**1.14**	(1.04-1.24)
Other cause	Pooled	18,852	491/229.1	**1.00 (ref.)**	**1.44**	(1.12-1.86)	**1.31**	(0.95-1.81)	**1.09**	(1.00-1.19)
	Czech Rep.	6,100	186/82.6	**1.00 (ref.)**	**1.46**	(0.93-2.27)	**1.49**	(0.89-2.47)	**1.05**	(0.93-1.18)
	Russia	6,474	153/64.2	**1.00 (ref.)**	**1.23**	(0.83-1.85)	**1.51**	(0.88-2.59)	**1.13**	(0.99-1.30)
	Poland	6,278	152/82.2	**1.00 (ref.)**	**1.28**	(0.82-2.00)	**1.00**	(0.57-1.78)	**1.04**	(0.91-1.19)

All HRs are adjusted for age, sex, country-cohort, energy intake, education, marital status, level of deprivation, smoking, alcohol intake, leisure-time physical activity, fresh fruit intake and fish intake
