# Supplementary material for: Traditional Eastern European diet and mortality: prospective evidence from the HAPIEE study
Source: Eur J Nutr. 2020 Jul 1;60(2):1091–100. doi: 10.1007/s00394-020-02319-9 (PMC7900332; doi:10.1007/s00394-020-02319-9)
Supplement: Supplementary file 2 — Supplementary file2 (PDF 609 kb) [file 394_2020_2319_MOESM2_ESM.pdf]

ONLINE RESOURCE 2: List of literature sources used to identify dietary habits of rural communities in the 1950s/1960s in selected Eastern European countries:

Manuscript title: Traditional Eastern European diet and mortality: prospective evidence from the HAPIEE study

Journal Name: European Journal of Nutrition

Authors: Denes Stefler, Daniel Brett, Eszter Sarkadi-Nagy, Ewa Kopczynska, Stefan Detchev, Aniko Bati, Mircea Scrob, Diane Koenker, Bojan Aleksov, Elodie Douarin, Galina Simonova, Sofia Malyutina, Ruzena Kubinova, Andrzej Pajak, Milagros Ruiz, Anne Peasey, Hynek Pikhart, Martin Bobak

Corresponding author: Denes Stefler

Department of Epidemiology and Public Health, University College London

Email: denes.stefler@ucl.ac.uk

BULGARIA

- Чолчева, П., Русева, Ал. (ред.): Книга за домакинята *[Book for the Housewife],* София: Издателство на Националния съвет на Отечествения фронт, 1956, 1957, 1958, 1959.
- Ковачева, А., Хаджиева, Ел, Гайдарова, М. (ред.): Книга за домакинята *[Book for the Housewife*], София: Издателство на Националния съвет на Отечествения фронт, 1962
- Ковачева, А., Хаджиева, Ел. (ред.): Книга за домакинята *[Book for the Housewife],* София: Издателство на Националния съвет на Отечествения фронт, 1966.
- Чолчева, П.: Съвременна готварска книга. [*Contemporary cookbook*] Трето допълнено издание. София: Техника, 1964, reprinted in 1966.
- Шопова, Ст. Бозукова, Л.: Готварска книга за столовете и горските стопанства, [*Cookbook for the Canteens and Forestry*], София: Комитет за горите и горската промишленост, Земиздат, 1965.
- Кръстева, Гина. Храна Народна храна и хранене – В: Добруджа. Етнографски, фолклорни и езикови проучвания [*Food and Foodways. Popular food and Food ways]* – In: Dobrudja. Ethnographic, folkloristic and linguistic studies, София: издателство на БАН, 1974, с. 249-261.
- Радева, Лилия. Храна и хранене – Пирински край. Етнографски, фолклорни и езикови проучвания [*Food and Foodways. Pirin Region. Ethnographic, folkloristic and linguistic studies].* София: издателство на БАН, 1980, с. 347-367.
- Радева, Лилия. Храна и хранене – В: Пловдивски край. Етнографски и езикови проучвания. [*Food and Foodways – In: Plovdiv region. Ethnographic and linguistic studies*] София: издание на Българската академия на науките, 1986, с. 166-187.
- Радева, Лилия. Храна и хранене – Ловешкия край. Материална и духовна култура [*Food and Foodways – In: Lovech region. Material and spiritual culture*]. София: Академично издателство „Марин Дринов“, 1989, с. 239-254.

CZECH REPUBLIC

- Franc M: Řasy, nebo knedlíky? Postoje odborníků na výživu k inovacím a tradicím v české stravě v 50. a 60. letech 20. století [*Algae, or Dumplings? Attitudes of nutrition experts on traditions and innovation in nutrition in the Czech lands in 1950s and 1960*s]. Praha: Scriptorium, 2003. pp. 119–130.
- Haukanes H: Ambivalent traditions: transforming gender symbols and food practices in the Czech republic. Anthropology of East Europe Review 21.1 (2003): 77-82.
- Haukanes H, Pine F: Ritual and everyday consumption practices in the Czech and Polish countryside: conceiving modernity through changing food regimes. Anthropological Journal on European Culture 12.1 (2004): 103-130.

HUNGARY

- Báti A: Régi és új elemek a cserépfalui konyhán [*Old and new elements in Cserépfalu`s kitchens*]. Budapest: Magyar Néprajzi Társaság [Hungarian Ethnographic Society]. 2008.
- Báti A: *Traditional food in modern Hungarian food culture.* In Zdenek Uherek et al (eds): Traditional Food in Central Europre. 119-129. Prague, 2013.

- Báti A: *The Return of Wood-fired Baking Oven in Hungary*. In: Patrcia Lysagth (ed.): The Return of Traditional Food. 118-128. Lund: Lund University Press. 2013.
- Fél E, Hofer T: Arányok és mértékek a paraszti gazdálkodásban [*Proportions and measures in the peasant economy*]. Budapest. Balassi kiadó. 1997. (Available at: *mek.oszk.hu/04900/04950/04950.pdf*)
- Kozponti Statisztikai Hivatal [Hungarian Central Statistical Office]: Az élelmiszerfogyasztás alakulása Magyarországon [*Changes of food consumption in Hungary*]. Statisztikai Időszaki Közlemények 6. Budapest. 1957.
- Kozponti Statisztikai Hivatal [Hungarian Central Statistical Office]: Étrendi szokások a munkás-, alkalmazotti- és paraszti háztartásokban [*Eating habits in the factory worker, employee and peasant households*]. Statisztikai időszaki közlemények 34. Budapest. 1960.
- Nyisztor T: Hétköznapok és ünnepnapok. A moldvai magyarok táplálkozásának ethnográfiája. [*Everydays and festive days. Ethnography of the dietary habits of Moldovan Hungarians*]. Cluj Napoca: Kriza János Néprajzi Társaság [János Kriza Ethnographic Society]. 2013.
- Valuch T: Rántott leves, cukros-zsíros kenyér és borjúbécsi [*Wienersnitzel*]. Országos Széchényi Könyvtár 1956-os Intézet és Oral History Archívum [National Széchényi Library 1956 Institute and Oral History Archive]. 2000. (Available at: http://www.rev.hu/ords/f?p=600:2:::::P2_PAGE_URI:valuch_borjubecsi)
- Valuch T: Magyar hétköznapok [*Hungarian weekdays*]. Budapest. Napvilág Kiadó. 2013.

POLAND

- Czerniewska M: Budżety domowe rodzin chłopskich [*Domestic budgets of peasant families*]. Państwowe Wydawnictwo Ekonomiczne, Warszawa. 1963.
- Koprowska H (ed.): Jadłospisy racjonalnego żywienia w stołówkach. [*Rational Daily Menus norms for state collective farms' eateries*]. 1966.
- Seweryn S, Bucholc M, Rygiel E (eds.): Zestawy obiadowe dla stołówek I [*Lunch menus for eateries*], zeszyt nr 13, Centralne Laboratorium Przemysłu Gastronomicznego, Warszawa. 1970.
- Statystyka Polski [Statistics Poland]: Budżety rodzin robotniko-chłopów 1967 [*Budget of peasant families*], Materiały Statystyczne nr 38. Warszawa. 1969.

ROMANIA

- Bucur B: Prolegomena to the Romanian sociology and historiography of food. International Review of Social Research 2017; 7(1): 57–68.
- Flaut D, Tusa E: Some aspects of social life in Romanian villages in the interwar period. In: Hoskova-Mayerova S, Maturo F, Kacprzyk J (eds.). Mathematical-statistical models and qualitative theories for economic and social sciences. Springer, Cham, 2017. pp 425-437.
- Scrob M: Developments in food consumption in socialist Romania during the 1960s and 1970s: implications for a re-evaluation of consumers' experiences under socialism. NEC-Odobleja Yearbook, Bucharest, 2018.

RUSSIA

- Caldwell ML. Not by bread alone: Social support in the new Russia. Univ of California Press, 2004.
- Glants M, Toomre J (eds.): Food in Russian history and culture. Bloomington: Indiana University Press, 1997.
- Glushchenko I. Obshchepit: Mikoian i sovetskaia kukhnia. [*Public catering: Mikoyan and Soviet cuisine*]. 2nd ed. Moscow: Izdatel'skii dom Vysshei shkoly ekonomiki, 2015.
- Honkanen, P, Voldnes G: Russian consumers' food habits. Results from a qualitative study in Moscow. Nofima rapportserie (2006).
- Jacobs AK: V.V. Pokhlebkin and the search for culinary roots in late Soviet Russia. Cahiers du monde Russe. Volume 54, Number 1-2, Jan-Jun 2013, pp. 165-186.
- Kaganova A.(ed.): Kulinariia. Leningrad: Gostorgizdat, 1959.
- Koenker DP: The taste of others: Soviet adventures in cosmopolitan cuisines. Kritika: Explorations in Russian and Eurasian History; Slavica Publishers; Volume 19, Number 2, Spring 2018, pp. 243-272
- Pokhlebkin VV: *Natsional'nye kukhni nashikh narodov: Osnovnye kulinarnye napravleniia, ikh istoriia i osobennosti. Retseptura.* [The national cuisines of our peoples: fundamental culinary trends, their history and particularities, with recipes.] Moscow: Pishchevaia promyshlennost', 1978.
- Scientific Research Institute of Nutrition of the Russian Academy of Medical Sciences: Историческая справка [*Historical Reference*] (Available at: http://www.ion.ru/index.php/2008-12-16-10-17-21)
- Sivolap IK (ed.): Kniga o vkusnoi i zdorovoi pishche [The Book of tasty and healthy food]. Moscow: Pishchepromizdat, 1953.
- Vail P, Genis A: Russian Cuisine in Exile. (Trans. Angela Brintlinger and Thomas Feerick.) Boston: Academic Studies Press, 2018.

VARIOUS COUNTRIES

- Kristbergsson K, Oliveira J (eds): Traditional foods. General and consumer aspects. Springer. New York, 2016.
- Lysaght P, Jönsson H, Burstedt A (eds): The return of traditional food. (Lunds Studies in Arts and Cultural Sciences; Vol. 1). Lund University, 2013.
- Pojarová T (ed): Traditional Food in the Central Europe. Prague: Institute of Ethnology of Czech Academy of Sciences, 2013.
- Weichselbaum E, Benelam B, Costa HS: EuroFIR synthesis report No. 6: Traditional Foods in Europe. EuroFIR Project Management Office/British Nutrition Foundation, 2009. (Available at: http://www.eurofir.org/wp-content/uploads/2013/09/Synthesis-Report-No-6.pdf)
